# Gynaeco-obstetrical follow-up of patients with dystrophic epidermolysis bullosa, a prospective study

**DOI:** 10.1186/s13023-024-03434-0

**Published:** 2025-03-21

**Authors:** M. Fournier, E. Bourrat, J. Rapp, D. Vexiau, C. Trastour, C. Chiaverini

**Affiliations:** 1 Dermatology Department, CRMR MAGEC Nord, Saint Louis Hôpital, Saint Louis APHP, Paris, France; 2 Dermatology Department, CRMR MAGEC Sud, Archet 2 University Hospital, Nice, France; 3 Gynaecology and Obstetrics Department, Archet 2 University Hospital, Nice, France

**Keywords:** Epidermolysis bullosa, Gynecology, Obstetrics, Sexually transmitted diseases, Cervical cancer, Breast cancer, Contraception

## Abstract

**Background:**

Dystrophic epidermolysis bullosa (DEB) is a rare group of genetic skin-fragility conditions resulting in blisters and erosions of the skin and mucosa, evolving into dystrophic and retractile scars. This study objective is to describe the genital involvement in women with DEB and their gynaeco-obstetrical life.

**Results:**

In this prospective two-centre study, data from women with DEB who were older than 18 years was gathered into a questionnaire by the dermatologist and/or gynaecologist investigators. This data was collected from patients’ medical records with regard to menstrual cycles, contraception methods, the obstetrical history, screening for gynaecological cancers and sexually transmitted diseases, and the sexual life. Vulvar examinations were carried out as part of the usual follow-up. In total, 27 women (median age 35 years; range 19 to 72) were recruited and their data included in the study between January and December 2021. The gynaecological follow-up was reported as regular for 14 of the 27 (52%) women; 16/27 (59%) mentioned menstruating; 13/24 (51%) had already had at least one contraceptive treatment; 17/27 (63%) declared they had sexual intercourse at least once, most often with difficulties; and 10/27 (37%) had full-term pregnancies, with 1 to 4 children per woman (i.e., 21 deliveries: 8 caesarean sections and 13 vaginal deliveries). Eleven of the 21 women who had a vulvar examination during follow-up had a lesion at the time of the exam. According to French recommendations, the rate of screening for sexually transmitted diseases (STDs), cervical cancer, and breast cancer was 18% (all over 25 years old), 70%, and 100%, respectively.

**Conclusion:**

As for all patients, women with DEB need gynaecological follow-up during their life. A sexology consultation is also highly recommended to help with the psychosexual aspect of DEB and to inform patients about specific preventive measures to avoid lesions during the sexual act, for contraception and for STDs screening. Pregnancies and deliveries are possible even in women with severe disease, most often without major complications.

**Supplementary Information:**

The online version contains supplementary material available at 10.1186/s13023-024-03434-0.

## Background

Dystrophic epidermolysis bullosa (DEB) is a rare group of genetic skin-fragility conditions resulting in blisters and erosions, most often posttraumatic but also inflammatory, that evolve into dystrophic and retractile scars. Mucosal involvement, particularly ear, nose and throat (ENT) and digestive involvement, are common and well described.

The description of genital involvement in women with DEB and their follow-up in Obstetrics and Gynaecology have been little analysed and constitute the subject of this study.

## Methods

In this two-centre (Archet 2 Hospital, Nice; Saint Louis Hospital, Paris) prospective study, data from women with DEB who were older than 18 years-old was gathered into our specific questionnaire by the dermatologist and/or gynaecologist investigators. This data was collected from patients’ medical records and covered menstrual cycles; contraception methods; the obstetrical history; screening for gynaecological cancers, sexually transmitted diseases (STDs) and breast cancer; and sexual life. The women’s referring dermatologist or gynaecologist performed vulvar examination as part of the usual follow-up. All women were verbally informed of the study, the study’s information note was given to them; All of them gave their consent; those actions are also filled in their medical record. The study is registered (reference R04-049) in the treatment registry of the Nice University Hospital under the declaration of conformity to the CNIL (the French Commission protecting the public’s privacy) to the reference methodology MR004. This protocol was registered at ClinicalTrials.gov (No. NCT04757727).

## Results

### Participants

In total, 27 women with DEB (median age 35 years, range 19–72 years) were included between January and December 2021. Patients had an intermediate subtype of DEB (n = 15, including 1 woman with dominant form and 10 with inversa recessive form) or a severe subtype (n = 12) (Table 1). Overall, 25 had mucosal involvement other than gynaecological: oral mucous membrane (n = 25), ENT (n = 21), oesophageal (n = 20) and/or ocular (n = 18) involvement. The mean body mass index (BMI) was 19.4 kg/m^2^ (data available for 17 of 27 women). Of these 17 women, 6 (all with generalized-severe subtype) had a BMI under 18.5 kg/m^2^ (i.e., malnourished), 14 had iron deficiency (severe subtype, n = 10; intermediary subtype, n = 4, including 3 patients with inversa subtype) and 11 chronic inflammatory syndrome (Table 1).

**Table 1:** Characteristics of patients (n = 27)

Age (years) Mean Median (IQR); range	41 35 (30–50); 19–72
Subtype of the disease Recessive severe Recessive inversa Recessive intermediate Dominant intermediate	12 10 4 1
Other mucosal involvement Oral mucous membrane Oesophageal Ocular ENT (Ear/nose/pharynx/larynx)	25 20 18 21
Deficiencies at the time of the questionnaire Iron deficiency Inflammatory syndrome Vitamin D deficiency Undernutrition (BMI < 18,5 kg/m^2^)	14 11 1 6/17 (data not available for 10 patients)
Gyneacological follow-up Have never seen a gynaecologist	17 10
Marital status Single In a relationship Unknown	14 12 1

*ENT* ear, nose, throat

### Gynaecological follow-up and screening

The gynaecological follow-up (gynaecological examination, information on and screening for gynaecological cancers) was regular for 14 of the 27 (51%) women, irregular (< 1 time per 2 years) for 3 (11%) and absent for 10 (37%) including 7 virgins. (Table 2, *larger than one A4 page, see at the end of the document text file; it should appear at the end of this paragraph*). Among the 10 women with no gynaecological follow-up, 6 (60%) had a severe RDEB form and 3 an inversa form; most were young (median age 33 years). These women justified the absence of gynaecological follow-up by their virginity (n = 2), the feeling of no need to be examined (n = 3), lack of time (n = 1), neglect (n = 1), fear of the examination (n = 1), and absence of orientation (n = 1), no answer (n = 1).

**Table 2:** Results by dystrophic epidermolysis bullosa (DEB) subtype

DEB type	Recessive severe (n = 12)	Recessive inversa (n = 10)	Recessive intermediate (n = 4)	Dominant intermediate (n = 1)
Iron deficiency	10/12	3/10	1/4	0/1
Undernutrition (BMI < 18,5 kg/m^2^)	6/10 (no data for 2 patients)	0/6 (no data for 4 patients)	0/2 (no data for 2 patients)	0/1
Anemia	9/12	2/10	0	0/1
Regular gynaecological follow-up: < every 2 years	6/12	5/10	3/4	0/1
Have never seen a gynaecologist	6/12	3/10	1/4	0/1
Irregular follow-up > every 2 years	0/12	2/10	0	1/1
Menstruations Yes and regular periods Yes and irregular periods No Causes	7/12 2/12 3/12 3/3: Estrogen plus progestin pill continuously or continuous progestin-only pill	4/10 0/10 6/10 3/6 Estrogen plus progestin pill continuously or continuous progestin-only pill 2/6: Menopause 1/6: Unexplained amenorrhoea	3/4 0/4 1/4 1/1: Menopause	0/1 0/1 1/1 1/1: Menopause
Sexual intercourse > 1 time/week > 1 time/month About 1 time/year Only one sexual intercourse Unknown intercourse frequency	5/12 1/5 1/5 2/5 1/5	8/10 0/10 6/8 1/8 0/8 1/8	3/4 0/3 2/3 0/3 0/3 1/3	1/1 0/1 0/1 0/1 0 1/1
Difficulty during intercourse (dyspareunia, stenosis, apprehension, sores)	3/5	7/8	0/3	No information
Women who had pregnancies Vaginal delivery Cesarean section	1/12 0/12 1/12	5/10 3/5 2/5	3/4 2/3 1/3	1/1 1/1 0/1
Lesion of the vulva at the time of the examination Examination not performed	5/12 2/12	3/10 1/10	3/4 0/4	0/1 1/1

*BMI* Body Mass Index

### Periods and protections

Sixteen of 27 (59%) women had menstruation, mostly regular, with variable severity (light, n = 2; moderate, n = 5; heavy, n = 6; no information for 3). Two women with a severe subtype had irregular menstruation due to undernutrition. Eleven of 27 (41%) no longer had periods due to menopause (n = 4), continuous use of oestroprogestative or progestin-only contraceptives (n = 6) and secondary unexplained amenorrhoea (n = 1), without undernutrition (Table 2). The method of protection was sanitary pads for all 16 women, which was well tolerated in 11 (69%).

### Contraception

Thirteen of 24 (54%) women had already undergone at least one contraceptive treatment; some had different contraception methods during their life, most of the time combined estrogen-progestin pill, continuously (n = 5/13; 38%) or sequentially (n = 2/13; 15%) but also continuous progestin-only pill (n = 4/13; 31%), intrauterine device (n = 3/13; 23%), or hormonal patch (n = 1/13; 0,07%) for a woman with an inverted subtype.

Four women took oral contraception only to prevent menstrual discomfort and aggravation of iron deficiency anaemia (intermediate inversa form, n = 2; generalized form, n = 2), but 2 women who had anaemia due to an iron deficiency rejected any anti-anaemia contraception.

### Sexual intercourse and privacy

Twelve of 27 (44%) women were in a relationship, 14 (52%) were single; family status was unknown for 1 (3%) (Table 1). Overall, 17 of 27 (63%) women had already had sexual intercourse at least once (9 at least once a month, 1 once a week and 3 once a year) (Table 2).

Ten of 17 (58%) women had difficulties during intercourse (for one or multiple reasons): 9 (56%) linked to pain during and/or after intercourse, 5 (31%) to anxiety, 4 (25%) to sores, 2 (13%) to post-coital bleeding, and 2 (13%) to vaginal stenosis.

Concerning the use of lubricant (type unspecified), 9 of 17 (53%) women were using it at least once: 3 (17%) regularly, 4 (23%) often, 2 (12%) occasionally and 4 (23%) never (unknown for 4)*.* Analysis according to DEB subtypes showed that among the 12 women with recessive severe form, only 5 (42%) had already had intercourse (less than once a year for 2 of them and only once for one) (Table 2), whereas among the 10 women with an inversa form (characterised by a major perineal injury), 8 (80%) were having sexual intercourse (at least once a month for 6), but with difficulty such as pain, sores, and anxiety for 7 (88%). Among the 4 women with a recessive intermediate, 3 had sexual intercourse (and all carried pregnancies). Sexuality information was not present for the woman with a dominant intermediate form but she had had one child by vaginal delivery.

### Obstetric history

Ten in 27 (37%) women had full-term pregnancies with 1 to 4 children per woman (i.e., 20 deliveries: 8 by caesarean section and 12 by vaginal delivery). Six women gave birth by vaginal delivery: 5 with a recessive intermediate form, including 3 with an inversa subtype and 1 with a dominant intermediate form (Table 3). The mode of delivery remained the same for each woman in each pregnancy. The median weight of the infants was 3.125 kg (range 1.68–4.3 kg). Three women had had complications during delivery, with no major complications: vulvar wounds (inversa form, n = 2) and wall hematoma (severe form, n = 1). Four women breast-fed right after their delivery: 3 had had complications (pain, cracks, erosions), which led them to stop breast-feeding, and 1 (intermediate form) had no difficulty over a long period.

**Table 3:** Patient delivery by DEB subtype (10 women)

DEB type	Vaginal delivery	Cesarian section	Total women by subtype
Recessive severe	0	1	1/12
Recessive inversa	3	2	5/10
Recessive intermediate	2	1	3/4
Dominant intermediate	1	0	1/1
Total	6	4	10/27

*DEB* dystrophic epidermolysis bullosa

### Vulvar injuries and gynaecological examination (Figs. 1, 2 and 3)

**Fig. 1:**
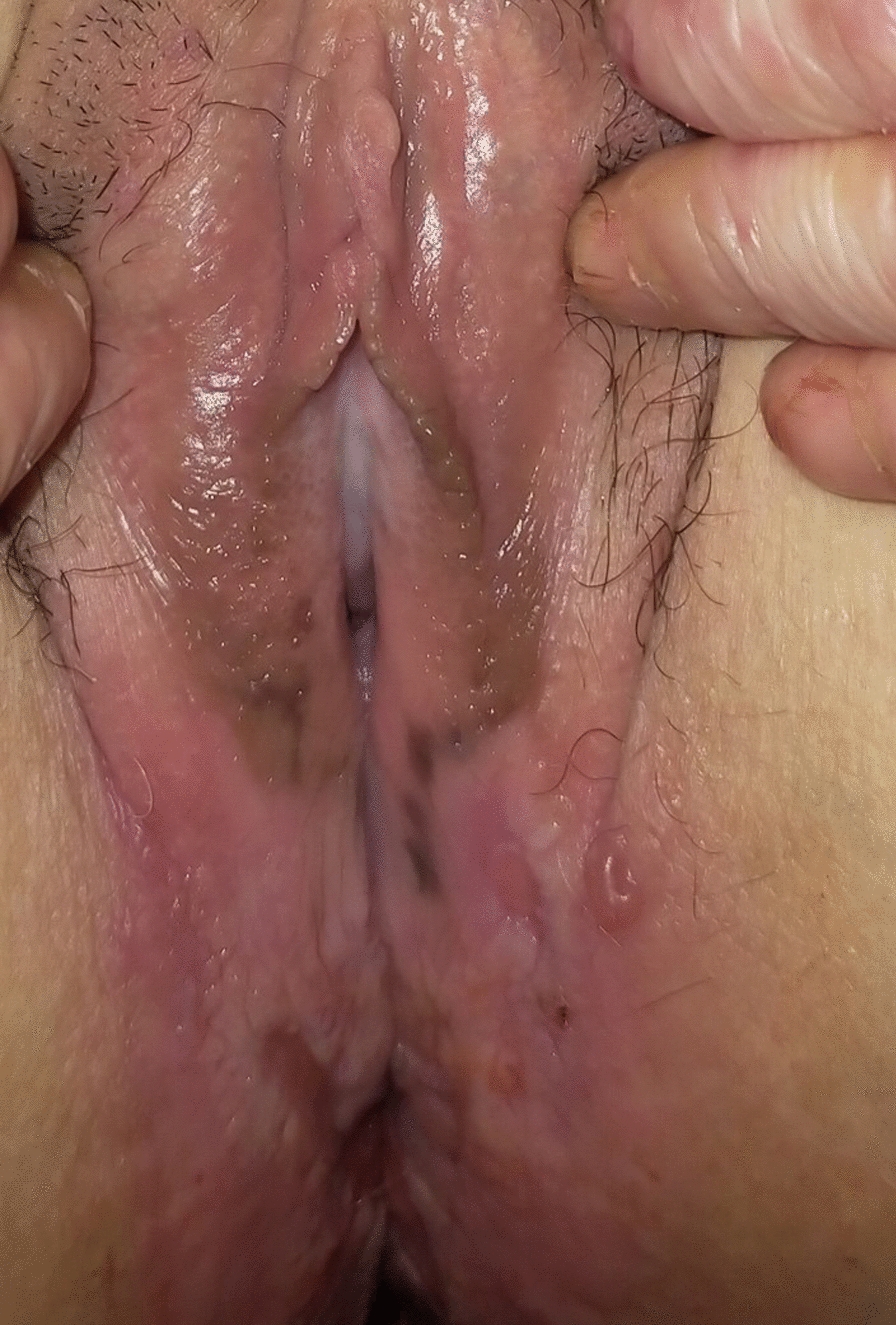
Blistering, pigmentation and chronic peri-anal erosion in a patient followed for dystrophic epidermolysis bullosa, a severe form

**Fig. 2:**
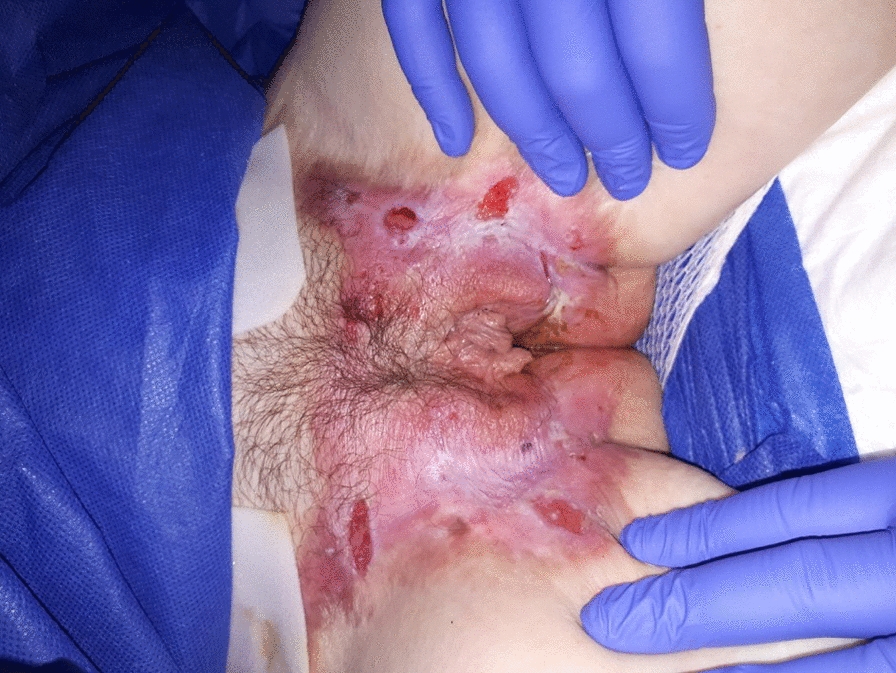
Sores, erosion and vulvar dystrophy in a patient followed for dystrophic epidermolysis bullosa, an inversa form

**Fig. 3:**
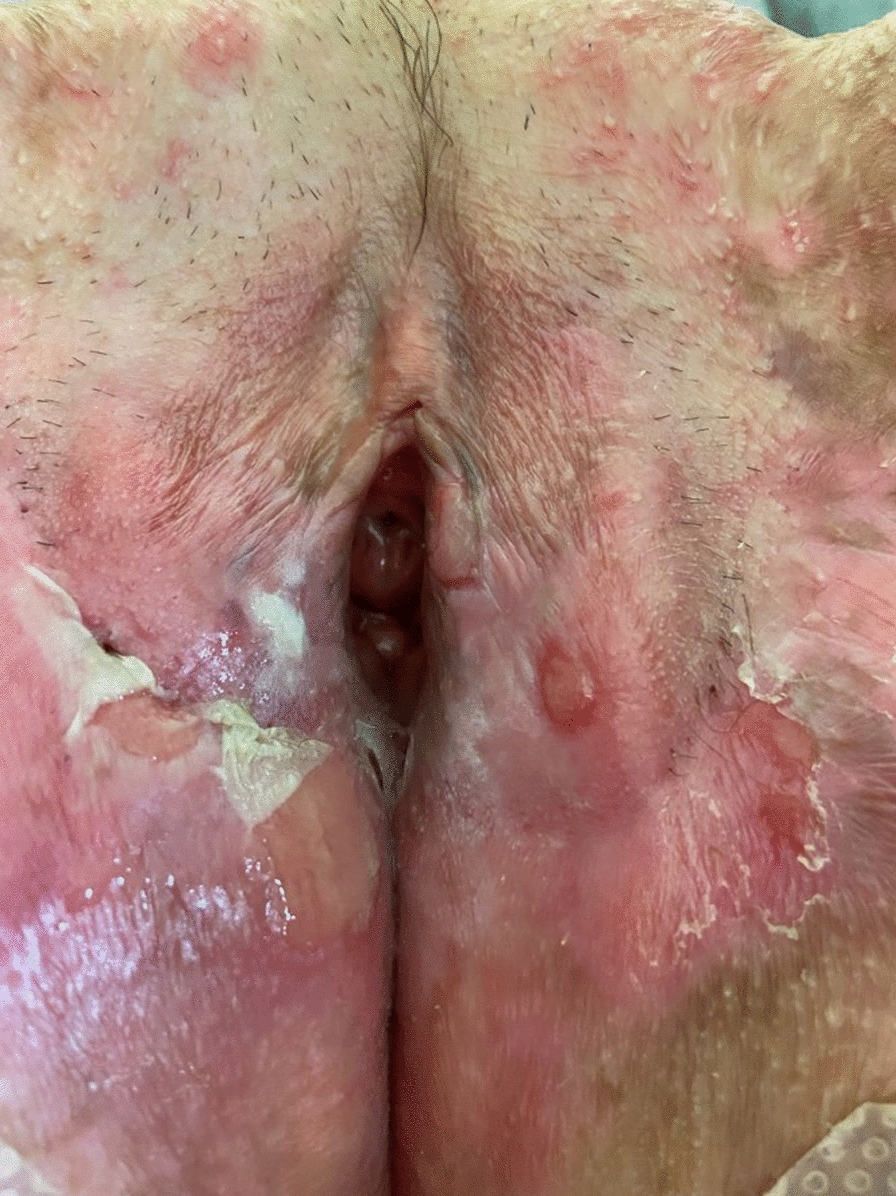
Blistering, erythema, erosions, desquamation and vulvar atrophy, in a patient followed for dystrophic epidermolysis bullosa

Overall, 21 of 27 (78%) women underwent a perineal examination that revealed vulvar lesions in 11 (52%): sores (n = 3), erosions (n = 3), pigmented lesions (n = 2) or hypopigmented lesions (n = 1), vulvar or anal dystrophia/atrophy (n = 6), vulvar stenosis (n = 4), condyloma (n = 1) and severe perianal erosions (n = 1). There was no bacterial infection.

We found vulvar involvement in 5 of 12 (42%) women for the recessive severe subtype, 3/10 (30%) for the recessive inversa subtype, and 3/4 (75%) for the other recessive intermediate subtypes (Table 2).

### Screening for STDs

The French health authority recommends [1] that all sexually active women aged 15 to 25 years be offered screening for *Chlamydia trachomatis* at least once. In our study, of the 17 women who had sexual intercourse at least once, 3 (18%), all over 25 of age, were offered a screening at least once in their life time (negative in 2 cases, not done in 1 case). One woman with a history of genital warts did not had a prescription for screening. No history of STDs was reported.

### Screening for cervical *cancer*

Seventeen of 27 (63%) women were under French recommendations for cervical cancer screening [2]; 12 (70%) had a cervical smear with 3 positive human papillomavirus (HPV) tests (one patient with DDEB, 2 patients with RDEB intermediate), including one with cervical dysplasia leading to conization surgery. Reasons for women to be unscreened (n = 5, RDEB intermediate n = 2, RDEB inversa n = 2, RDEB severe n = 1) were fear of pain (n = 1), few intercourses (n = 1), lack of gynaecological follow-up (n = 1) and no information (n = 1). The reason was unknown for one patient.

### Screening for breast *cancer*

The French health authority [3] recommends an annual mammography for women aged 50 to 74 years. In our study, all 7 women concerned had a mammography or MRI (in case of severe skin fragility) with a minimum follow-up every other year. Three women under 50 years old benefited from screening by mammography (n = 1) or echography (n = 2).

## Discussion

The improved management of DEB in recent years has led to an increase in life expectancy and improved medical condition. As a result, young girls become young women with periods and the possibility and the desire of having sexual relations and/or procreation. This scenario requires prevention and education, as recently recommended in evidence based guidelines for sexuality [4], pregnancy, childbirth and aftercare [5] in EB. Dermatologists, who are often at the heart of the multidisciplinary management, must be sensitized to and trained in these points to inform and guide women effectively. The gynaeco-obstetrical life of women with EB is thus a new subject of study in the EB domain, with still few articles published; mainly focused on obstetrics [6–9]. Intong et al. [7] in particular, have shown in a prospective study that most patients with EB are capable of giving birth without increased risk of pregnancy-related complications including women with DEB. The originality of our study was its systematic approach to all aspects of the gynaecological life of women with DEB, including prevention and obstetrics.

The first point of interest is that only 17 of 27 women had gynaecological follow-up, regular for 14 and irregular for 3. Therefore, 10 women had no gynaecological follow-up despite being cared for in an EB reference centre. Only one woman had not been referred to a gynaecologist. The other women did not feel concerned (n = 5, including 2 virgins) or preferred to avoid or postpone this follow-up (n = 4), because of fear for at least one of them. This situation underlines the importance of giving information about the need for regular follow-up (including cycle monitoring, contraception, sexuality information, prevention of STDs, women’s cancers and obstetrical monitoring) and cooperation with a gynaecologist trained in the skin-mucosal peculiarities of DEB to reassure women and avoid traumatic examinations.

We found no data in the literature about menstruation in women with DEB. Our study confirms our perception that menstruation in these women is identical to that in the female population of equivalent age, provided that BMI is preserved (very underweight women with DEB often have secondary amenorrhoea). Pads appear to be the main means of protection and are well tolerated in most cases. According to French recommendations [10], DEB women with anaemia due to a martial deficiency and abundant menstrual flux should be on non-stop contraception with an anti-anaemic aim. Indeed, in our study, one-third of the women taking contraceptives had this indication, but 2 women rejected it. Several kinds of contraception could be offered to these women (intrauterine device (with progestin or without); combined estrogen-progestin oral pill or progestin pill/ patch, provided that the skin condition allows it). For women with oesophageal stenosis or a gastrostomy tube, contraceptive pills melt easily in the mouth. No thromboembolic episodes were reported, even in patients with chronic inflammatory syndrome. Finally, most women (80%) who became pregnant were previously on contraception. This underlines the importance of patient information for the interest of contraception to limit iron deficiency, its good tolerance and the lack of negative impact on future fertility.

Concerning sexuality, no woman in our study had reported having had a sexology consultation during follow-up, which demonstrates the need for further work in this area because of the important role of psychosexual factors in the altered quality of life of women with DEB [11]. However, despite their mucosal fragility, more than half of the women in our series had already had sexual intercourse, most often complicated by sores, erosions and pain, the discomfort for sexuality depending on the degree of damage. This finding agrees with the literature emphasizing that being diagnosed with DEB does not deny or hamper the wish or capacity of an individual to participate in sexual activities [4], but women with DEB have psychosexual difficulties that affect their quality of life and dyspareunia [12].

The number of women who had a sexual experience was lower for those with a severe RDEB form (42%) than an inversa form (80%). The altered general state and the very damaged body image of the women with severe RDEB (mitten-like hands, diffuse wounds and dressings, alopecia, poor dental condition), which harms sexuality, are more important factors than the state of the perineum, which is probably worse in the inversa than severe forms.

In total, 9 of 17 (52%) women had used lubricant at least once during sexual intercourse, but the subtype was not specified. We stress the importance of informing women using condoms that condoms lose their effectiveness when in contact with certain lubricants such as petroleum jelly (which makes the latex porous to germs and spermatozoids). Water or silicon-based lubricants may be useful to minimize friction and preserve the mucous membranes [4, 5, 12]. They can also use no latex condoms which aren’t porous so that they can be used with lubricant and also with local oestrogens therapy. In a recent publication, King et al*.* [4] proposed sexuality guidelines for women with EB. However, the authors had few data from the literature to construct reliable recommendations. Our results are the first to deal with those topics. Finally, although more than half of the women had had sexual intercourse at least once, screening for HPV infection/cervical cancer and STDs *(chlamydia/gonococcus)* is not sufficiently prescribed by referring physicians, which suggests that physicians may underestimate the sexual activity of their patients. Indeed, these data oppose the excellent rate of breast cancer screening in women over age 50 years, who have less severe forms, by mammography or MRI.

In a small case series of 6 women with EB, including 3 with DEB, Dang et al*.* [12] showed that 5 of 6 women were affected by vulvar and anal involvement. In our study based on 21 women with DEB and vulvar examination, perineal involvement was common in post-pubescent women, in all DEB forms, and was both acute, due to mucosal fragility, such as sores or erosions, but also chronic, linked to scaring of repeated traumatisms, with atrophy, synechiae, dyschromia and vulvar stenosis. However, in contrast to Dang et al*.*, [13] we found only a few women with anal involvement: one with both severe acute and chronic lesions and one with anal atrophic lesions. No vulvar epidermoid carcinoma was identified, but in a systematic review of cutaneous epidermoid carcinoma in EB patients, one case of vulvar involvement and one case of anal involvement were reported, which suggests mandatory screening for perineal cancer in women with DEB.

Women with DEB often worry about pregnancy and much more about childbirth. However, in the few studies, concerning pregnant women with DEB, no major antenatal obstetric complications during pregnancy were noted [6–8, 14–17], with correct cervico-vaginal and perineal tolerance, regardless of the delivery type [5, 7]. Recommendations on pregnancy, childbirth and aftercare in EB were recently published [5] to help physicians care for these patients. The natural way seems the best for delivery [5–7, 9, 12, 14, 15], provided there is no severe damage on the genital tract (stenosis) and that genital mucosa in DEB can withstand vaginal delivery [15]. The mother must be involved in the decision about the delivery type and physicians should encourage women with EB to prepare a birth plan [5]. Our data agree with these recommendations. Delivery is also a real challenge for anaesthetists who have to follow specific recommendations tailored to DEB patients [5, 17–19]. Choosing a specialized maternity hospital will depend on the degree of EB severity in the mother and/or the child to be born [5]. The median weight at birth is near the French national median (3.128 kg in our study vs 3.264 kg for the French median weight in 2021) [20].

As usual, when dealing with a chronic disease, the nutritional state must be strengthened and the anaemia checked before attempting pregnancy, with supplementation when necessary. Furthermore, before any pregnancy project, the couple must be well informed of the risks of transmission and the possibilities of anterior or preimplantation diagnosis if necessary; this situation implies a precise and certain diagnosis (if possible molecular) of the type and subtype of DEB.

In our study, 4 of 10 (40%) women breast-fed after birth, but for most, it was difficult (skin cracks, erosions), which led to stopping breast-feeding for 3 of the 4 women. Indeed, this finding fits with the literature, which mentions that nipple blistering is frequent for these women [5–8, 15, 17]. Mothers could be advised on how to position the infant’s mouth properly on the part of the areola where the skin is harder. One could also propose how to place nipple protectors [6].

The limits of this study are the restricted number of patients (n = 27) and that women were only followed-up in reference centres for DEB (Nice and Paris), with a majority of recessive severe or inversa forms of the disease. We did not use a quality-of-life questionnaire to rate the impact of vulvar involvement and difficulties in emotional and sexual lives. However, among the women who never had intercourse, most reported that this was linked to their condition.

## Conclusion

Women with DEB need a gynaecological follow-up, including perineal examination, during their life, as for every woman. Because most DEB women have sexual intercourse, a sexology consultation is also highly recommended to help with the psychosexual aspect of DEB and to inform patients for preventive specific measures to avoid lesions during the sexual act, for contraception and for STDs infection/screening. Pregnancies and deliveries are possible even in women with severe disease, most often without major complications; however breastfeeding is often difficult. Gynaecologists and obstetricians must be trained to meet the specific needs of these women.

## Supplementary Information

Additional file1.

## Data Availability

All data generated or analyzed during this study are included in this publishedarticle and its Supplementary table file.

## Abbreviations

ENT: Ear, nose and throat
BMI: Body mass index
STDs: Sexually transmitted diseases
DEB: Dystrophic epidermolysis bullosa
EB: Epidermolysis bullosa
MRI: Magnetic resonance imaging
HPV: Human papillomavirus
